# The Molecular Basis of Wound Healing Processes Induced by Lithospermi Radix: A Proteomics and Biochemical Analysis

**DOI:** 10.1155/2012/508972

**Published:** 2012-09-17

**Authors:** Chia-Yen Hsiao, Tung-Hu Tsai, Kin-Fu Chak

**Affiliations:** ^1^Institute of Biochemistry and Molecular Biology, School of Life Sciences, National Yang-Ming University, Taipei 11221, Taiwan; ^2^Institute of Traditional Medicine, National Yang-Ming University, Taipei 11221, Taiwan; ^3^Department of Education and Research, Taipei City Hospital, Taipei 11221, Taiwan; ^4^Department of Medicine, Mackay Medical College, Taipei 25245, Taiwan

## Abstract

Lithospermi Radix (LR) is an effective traditional Chinese herb in various types of wound healing; however, its mechanism of action remains unknown. A biochemical and proteomic platform was generated to explore the biological phenomena associated with LR and its active component shikonin. We found that both LR ethanol extracts and shikonin are able to promote cell proliferation by up to 25%. The results of proteomic analysis revealed that twenty-two differentially expressed proteins could be identified when fibroblast cells were treated with LR or shikonin. The functions of those proteins are associated with antioxidant activity, antiapoptosis activity, the regulation of cell mobility, the secretion of collagen, the removal of abnormal proteins, and the promotion of cell proliferation, indicating that the efficacy of LR in wound healing may be derived from a synergistic effect on a number of factors induced by the herbal medicine. Furthermore, an animal model confirmed that LR is able to accelerate wound healing on the flank back of the SD rats. Together these findings help to pinpoint the molecular basis of wound healing process induced by LR.

## 1. Introduction 

 Lithospermi Radix (LR, the dried root of *Lithospermum erythrorhizon* Sieb. et Zucc., also called Zicao or Gromwell) is commonly used to treat skin disorders such as cuts and burns. LR is one of the five components (*Angelica *Radix, Lithospermi Radix, oleum sesame, cera flava, and adeps suillus) of Shiunko, a traditional wound healing herbal medicine that has been used for several hundred years in China [1]. A recent study showed that LR has multiple activities including antimicrobial activity [2], antiviral activity [3], anti-inflammation activity [4], and antitumor activity [5]. The most important components in LR are derivatives of shikonin, such as deoxyshikonin, shikonin, acetylshikonin, isobutylshikonin, and others [6]. 

 Herbal drugs are difficult to analyze because of their complexity and toxicity. In order to circumvent these problems, recent research has been dominated by analysis of the major or most effective single component in individual herbal medicines, for example, *Astragalus* saponins (AST) from *Astragalus membranaceus* [7]. However, the effects of a total herbal extract and its most active component are not necessarily the same. In addition, a previous study by us demonstrated that the efficacy of *Angelica sinensis* total extracts in the wound healing process was significantly better than that of its active component, ferulic acid [8]. Furthermore, based on proteomic clustering, it was found that, in addition to the common group of proteins induced by *Angelica sinensis* total extract and ferulic acid, the total extract of *Angelica sinensis* could also induced some more specific proteins that may be of benefit for wound care.

 Proteomics is a powerful tool that has been widely used to analyze the complexity of protein changes in a biological system. Tsai et al. [9] found that acidic fibroblast growth factor (aFGF) was involved in the repair processes during spinal cord injury. Similarly, Sundaramurthi et al. [10] identified six proteins stimulated by Gastrodia elata blume (tianma) that may be useful as a remedy for neurodegenerative diseases.

 LR is a prominent herb that can be used alone or formulated with other drugs, such as Shiunko (formulated with *Angelica sinensis*) for the clinical treatment of skin disorders or skin trauma. Although LR does improve wound healing, the mechanism still remains to be resolved. In this work, a proteomics platform was used to explore the differences in the expression profiles of fibroblast cell proteins induced by an ethanol extract of LR or with shikonin. Based on these findings, a hypothetical mechanism for wound healing induced by LR is proposed. Furthermore, the efficacy of LR in wound healing was also verified using a SD rat model system. Thus, this work demonstrated that a proteomics approach is a suitable technological platform for the study of a complex traditional herbal medicine. In addition, the present work provides insights into the molecular impact of LR during the wound healing process. At the same time, the identified differentially expressed proteome may be useful as biomarkers in the future to monitor the efficacy of the drug in the wound healing processes. 

## 2. Materials and Methods

### 2.1. HPLC Analysis

The HPLC system was equipped with BAS PM-80 pumps, a DGU-20A5 degasser, a CMA/170 autosampler, and a Varian (model 340) photo-diode array detector. Chromatographic separation was performed using a Phenomenex Fusion RP-80 (250 × 4.6 mm, 4 *μ*m). The mobile phases were acetonitrile (solvent A) and 2% acetic acid (solvent B). For the analysis of LR, the mobile phase used the following elution gradient: 40% A to 90% A over 0–30 min at a flow rate of 1.0 mL/min with the detection wavelength set at 520 nm. The sample injection volume was 20 *μ*L.

### 2.2. Preparation of Plant Extracts

Dry roots of LR were bought in qualified traditional Chinese medicine store. It was smashed and incubated with 95% ethanol at 60°C for 30 min and this procedure was repeated 3 times. The mixture was then filtered by gauzes and filter paper, and the filtrates were retained. The filtrates were filled in a brown flask and concentrated by vacuum evaporator until dried. LR extracts were scraped and collected in bottles for further drying at 50°C overnight. The dried product was then stored in −20°C for further use.

### 2.3. Cell Culture

The human embryonic skin fibroblasts used in this study were Bioresource Collection and Research Center (BCRC) cell line number 60118 (Detroit 551) and the cells were cultured in 10 cm culture dish (Corning) with minimum essential medium (MEM alpha-modification, M0644, Sigma) containing 2 mM L-glutamine, 1.5 *μ*g/L sodium bicarbonate, 0.1 mM nonessential aminoacids, 1.0 mM sodium pyruvate, and supplemented with 10% fetal bovine serum (SAFC Bioscience), 50 units/mL penicillin, 0.05 mg/mL streptomycin, and 0.1 mg/mL neomycin (Sigma) in a humidified atmosphere at 37°C with 5% CO_2_. For passage, the cells were treated with 1 mL trypsin (2 mg/mL EDTA and 5 mg/mL trypsin in PBS) for 2 min, collected in a 50 mL tube (Corning), and then centrifuged at 1300 rpm for 5 min. The supernatant was discarded, fresh culture medium added, and finally the cells were dispensed into several 10 cm culture dishes.

### 2.4. Cell Viability Assay (WST-1 Assay)

The cell viability assay was performed according to the procedure described in the WST-1 manufacturer's manual (Roche) with minor modifications. The cells were seeded at 4 × 10^4^ cells/well (about 40% confluence) in 1 mL of culture medium into a 24-well culture plate and incubated at 37°C in an incubator containing 5% CO_2_. After cell attachment, the cultures were treated with either 0.5% DMSO as the control or the designated concentrations of LR or shikonin, which were added for 24 h. The supernatants were then discarded and 1 mL of WST-1 was added to each well at a 1 : 50 ratio formulated with fresh culture medium. The cells were then incubated for an additional 1 h in the dark. Finally, the absorbance was measured at 450 nm with the background being measured at 690 nm. The difference in absorbance was used to indicate the relative cell viability compared to the control treated with 0.5% DMSO. The experiments were performed using three replicates for each sample and the Student's  *t*-test was used for statistical significant analysis.

### 2.5. Sample Preparation for 2D PAGE

Fibroblasts were washed twice with phosphate-buffered saline (PBS) and lysed with 1 mL of NP-40 lysis buffer containing 10 mM Tris-HCl (pH 7.5), 50 mM NaCl, 1% NP-40, 30 mM Na_2_P_2_O_7_, 30 mM NaF, 1 mM Na_3_VO_4_, 1% protease inhibitor, and 1% phosphatase inhibitor (Sigma). The lysate was then scraped off, collected in an Eppendorf tube, and stored on ice for 1 h. The whole cell lysate was next centrifuged at 14000 rpm for 20 min at 4°C to remove insoluble material. The supernatant was transferred to a concentrator (GE Health, Vivaspin 20, 100 k MWCO) and centrifuged at 6000 ×g and 4°C until the volume was less than 500 *μ*L. At this point the lysis buffer was replaced with dH_2_O containing 1% protease inhibitor and 1% phosphatase inhibitor and the protein concentration quantified by Bradford protein assay (Bio-Rad). The lysate was then used directly for 2D PAGE analysis.

### 2.6. Analysis of the Protein Profiles Using 2D PAGE

The 2D PAGE was performed according to the method described in the manufacturer's manual (Amersham Biosciences) with minor modifications. For the first-dimensional IEF, pH 4–7, IPG strips (18 cm, BioRed) were rehydrated with 350 *μ*L rehydration buffer (0.5% IPG buffer, 8 M urea, 2% CHAPS, 50 mM dithiothreitol (DTT) and a trace bromophenol blue) for at least 3 h before a 100 *μ*g protein sample was loaded by cup loading. IEF was then carried out under the following conditions: 150 V for 5 h step-and-hold, 500 V for 3 h step-and-hold, 1000 V for 7 h gradient, 8000 V for 3 h gradient, and 8000 V for 18 h step-and-hold. For the second-dimensional SDS-PAGE, the IPG strips were equilibrated with 4 mL of equilibration buffer, containing 50 mM Tris-HCl pH 8.8, 6 M urea, 2% SDS, 30% glycerol, 50 mM DTT, and 0.01% bromophenol blue at room temperature for 15 min, which was followed by equilibration in 50 mM Tris-HCl pH 8.8, 6 M urea, 2% SDS, 30% glycerol, 5% iodoacetamide, and 0.01% bromophenol blue at room temperature for 15 min. The second-dimensional SDS-PAGE used a 12.5% separating gel and was performed without a stacking gel. Electrophoresis was carried out at 35 mA/gel until the tracking dye reached the bottom of the gel. The 2D PAGE was then stained by silver staining. 

### 2.7. Fast Silver Staining

After 2D PAGE electrophoresis, the gel was removed, immersed in fix solution (40% methanol and 10% acetic acid in dH_2_O) for 10 min, and then washed with dH_2_O for 10 min twice. After removal of the dH_2_O, and the gel was immersed in solution A (0.25 mM sodium thiosulfate) for 30 min, which was then replaced with dH_2_O for 10 min. After dH_2_O was removed, the gel was immersed in solution B (3.5 mM silver nitrate) for 30 min. After the gel was rinsed with dH_2_O, solution C (0.283 M sodium carbonate) and solution D (4.37 mM formaldehyde) in dH_2_O were mixed and used to develop the protein spots on the 2D PAGE gel. Finally, the reaction was stopped with 5% acetic acid.

### 2.8. Detection and Quantitative Analysis of the 2D PAGE Gels

2D PAGE images were captured and the amount of protein in each spot analyzed using ImageMaster 2D Elite software Version 5.0 (Amersham Biosciences). The volume of a protein spot was defined as the sum of the intensities of the pixel units within the protein spot. To correct for quantitative variations in the intensity of the protein spots, spot volumes were normalized as a percentage of the total volume of all the spots present in a gel.

### 2.9. Protein Identification by LC-MS/MS

The process is described in a document from the Institute of Biological Chemistry, Academia Sinica Institute of Biological Chemistry (http://proteome.sinica.edu.tw/) and this procedure was used with minor modification. Selected silver-stained protein spots were excised from the 2D PAGE. They were then desilver-stained using 1 : 1 Na_2_S_2_O (0.1 g in 1 mL H_2_O) and K_6_Fe(CN)_6_ (0.1 g in 1 mL H_2_O) in 25 mM ammonium bicarbonate until transparent. Next, 25 mM ammonium bicarbonate pH 8.5 was added to the gel slices for 10 min, which was followed by vacuum drying and rehydration with 50% acetonitrile in 25 mM ammonium bicarbonate pH 8.5 at room temperature for 10 min. The supernatant was then discarded and the gel slices vacuum dried again. Subsequently, the protein within the spot was trypsinized with 0.1% sequencing grade modified trypsin (Promega, Madison, WI, USA) in 25 mM ammonium bicarbonate, pH 8.5, at 37°C for at least 16 h. The supernatant was then transfered to a new Eppendorf tube, which was stored. The gel slices then had fresh 25 mM ammonium bicarbonate pH 8.5 added and the mixture sonicated for 1 min, which was repeated 10 times. The supernatant was then added to the previous supernatant in the stored Eppendorf tube. This was repeated by adding another aliquot of 50% acetonitrile in 25 mM ammonium bicarbonate pH 8.5 to the gel slices, which were sonicated again for 1 min repeated 10 times. This third supernatant was then added to the two previous supernatants and the pooled solutions evaporated to dryness under vacuum; the dried pellet was then separated on an integrated nano-LC-MS/MS system (Micromass) (National Research Program for Genomic Medicine, Academia Sinica). In addition, two low-level differentially regulated protein spots were identified by LC-MS/MS (Orbitrap) mass spectrometry system (Proteomics Research Center, National Yang-Ming University). The nano-LC-MS/MS data acquisition was carried out by Micromass ProteinLynx Global Server (PGS) 2.0 data processing software in the default mode and outputted as a single Mascot-searchable peak list (.pkl) file. The LC-MS/MS (Orbitrap) dataset was processed by SWQUEST (Thermo Finnigan). The peak list files were used to query the Swiss-Port version 2010_05 database using the Mascot program version 2.2 (release date, 28 Feb., 2007, Matrix Science, London, UK) with the following parameters: taxonomy, *Homo sapiens* (20,400 sequences), a peptide mass tolerance of 50 ppm, and a MS/MS ion mass tolerance of 0.25 Da. Only significant hits as defined by Mascot probability analysis were considered. Protein identifications were accepted with a statistically significant Mascot protein search score ≥36 or SEQUEST score = 2.5 (critical), which corresponds to an error probability of *P* < 0.05 using our dataset. The protein identification with the highest score was selected to eliminate protein redundancy within the database. 

### 2.10. Cluster Analysis and Functional Classification of the Differentially Expressed Proteins

A plot of the calibrated intensity of expression of each protein, as measured by the ImageMaster 2D Elite software Version 5.0 (Amersham Biosciences, Sweden) among the different groups of samples, was used in conjunction with an average linkage hierarchical clustering algorithm (UPGMA, Unweighted Pair Group Method with Arithmetic Mean); this was done using Hierarchical Clustering Explorer 3.5 [9]. The uncentered Pearson's correlation coefficient was determined as a measure of the similarity metric and the threshold value for the minimum similarity was set at 0.8. After clustering, each protein was allocated a place in a global temporal classification color heat map. We used BGSSJ (Bulk Gene Search System for Java; http://bgssj.sourceforge.net/) [9] and the Swiss-Prot protein knowledge database to carry out a functional classification of the proteins.

### 2.11. Western Blotting

Proteins extracts from fibroblast were separated by 12.5% SDS-PAGE and then transferred onto a nitrocellulose (NC) membrane. The NC membrane was blocked with 5% nonfat milk in TBST at room temperature for 1 h and probed with various different primary antibodies (anti-p-Erk, 1 : 1000; anti-Erk, 1 : 5000; anti-PRDX2, 1 : 5000; anti-p-p38, 1 : 1000; anti-p38, 1 : 1000 (from Cell Signaling); anti-LEG1, 1 : 500, Abgent; anti-TGF-*β*, 1 : 1000 (from Santa Cruz); anti-GSTP1, 1 : 1000; anti-GAPDH, 1 : 5000 (from GeneTex)) in 5% BSA in TBST. After washing, the NC membrane was treated with HRP-conjugated secondary antibody (Santa Cruz). The bands were visualized using chemiluminescent substrate (Millipore) and a chemiluminescent imaging system (LAS-4000, Fujifilm). The results are expressed as mean ± standard deviation. Student's  *t*-tests were performed to evaluate the statistical significance of any differences and a  *P*  value < 0.05 was regarded as statistical significant (*n* = 4 for each experiment).

### 2.12. Intracellular ROS (Reactive Oxygen Species) Assay

To measure the ROS content of the fibroblasts after treatment with 0.5% DMSO, LR5, LR20, or 100S, the intracellular H_2_O_2_ content was determined using the redox-sensitive fluorescent dye 2′,7′-dichlorofluorescein diacetate (DCF-DA) (Sigma). Briefly, the cells were cultured to confluence and trypsinized. After centrifugation, the supernatant was discarded and the cells resuspended and incubated with 10 *μ*M DCF-DA (20 mM in DMSO for stock solution and stored in −20°C) for 10 min at 37°C in the dark. The samples were then centrifuged, washed, and resuspended in fresh culture medium. A total of 8 × 10^4^ cells were added to a black flat 96-well ELISA plate with 200 *μ*L of medium. The relative concentration of intracellular ROS was determined by fluorescence reader using excitation at 485 nm and emission at 538 nm. Student's  *t*-tests were performed to evaluate the statistical significance of any differences and a  *P*  value < 0.05 was regarded as statistical significant (*n* = 3 for each experiment).

### 2.13. Boyden Chamber Migration Assay

After fibroblasts had been cultured to confluence, the cells were trypsinized, centrifuged, and resuspended in culture medium. A total of 2 × 10^4^ fibroblasts were then seeded in a Transwell (24 wells, Corning) after treatment with 0.5% DMSO, LR5, LR20, or 100S. These were then inserted into culture medium without bubbles and 6 h later the medium was removed from the Transwell and the cells fixed using methanol. After 10 min, the methanol was discarded and the fixed cells air dried. The cells were then stained using 5% Giemsa (solved in dH_2_O) at room temperature overnight. The Transwell was next washed by dH_2_O and the inner cells removed by rubbing with a cotton swab. The number of cells that had migrated through the Transwell was then counted manually by microscope. Student's  *t*-tests were performed to evaluate statistical significance of any differences and a  *P*  value < 0.05 was regarded as statistical significant (*n* = 3 for each experiment).

### 2.14. Wound Healing Assay

A total of 2.5 × 10^4^ fibroblasts were seeded on both sides of a culture insert (Ibidi) in a 24-well plate to generate a 500 m ± 50 m gap between the cells before drug treatment. After the cells were attached, the culture insert was removed carefully and the cells treated with each drug separately, which were added in culture medium. The cells were then incubated for 24 h. At this point the medium was discarded and the cells washed with PBS twice to remove any unattached cells. Finally, photographs were captured by digital camera under a microscope (Olympus) and wound healing measured. Student's  *t*-tests were performed to evaluate statistical significances of any differences and a  *P*  value < 0.05 was regarded as statistical significant (*n* = 3 for each experiment).

### 2.15. Sircol Collagen Assay

The process is described in the Sircol collagen assay general protocol (Biocolor). In brief, 3 mL cell medium were collected after treatment, and the collagen present was precipitated by 4 M NaCl. The collagen pellet was collected by centrifugation at 15000 ×g for 10 min at room temperature and then the pellet was redissolved in 0.5 mL of 0.5 M acetic acid. Sircol dye reagent was mixed with redissolved collagen sample at a ratio of 1.0 : 0.1 mL and the mixture gently inverted at room temperature for 30 min. The resulting collagen was collected by centrifugation at 10000 ×g for 10 min at room temperature. After discarding the supernatant, the pellets were redissolved in 1 mL of alkali reagent, and the collagen concentration determined at OD 540. Student's  *t*-tests were performed to evaluate statistical significances between any differences and a  *P*  value < 0.05 was regarded as statistical significant (*n* = 3 for each experiment).

### 2.16. Animal Experiment Protocol

SD rats were divided into four groups, namely, mock, DMSO, LR5, and LR20 with each group containing 6–8 individuals. After animals were anesthesized by zoletil (Virbac), their hair was shaved as clean as possible and their skin was sterilized using 70% ethanol. Two circular wounds of full thickness were generated using an 8 mm biopsy punch (World Precision Instruments, WPI) on the upper back of each SD rat. The diameters of wound size were between 7.5–9 mm. After surgery, each wound was treated twice per day with 0.5% DMSO, 5 *μ*g/mL LR, or 20 *μ*g/mL LR dissolved in 2% CMC (Sigma). The minimal diameters of each wound were measured twice per day, and the wounds were recorded by digital camera every 4 days. ANOVA was performed to evaluate statistical significances of the contraction of the wounds. The asterisks means of the wound size of both LR5 and LR20 were significantly smaller than DMSO, and  *P*  value was <0.01.

## 3. Results

### 3.1. Effect of LR and Shikonin on Cell Viability

**Figure 1:**
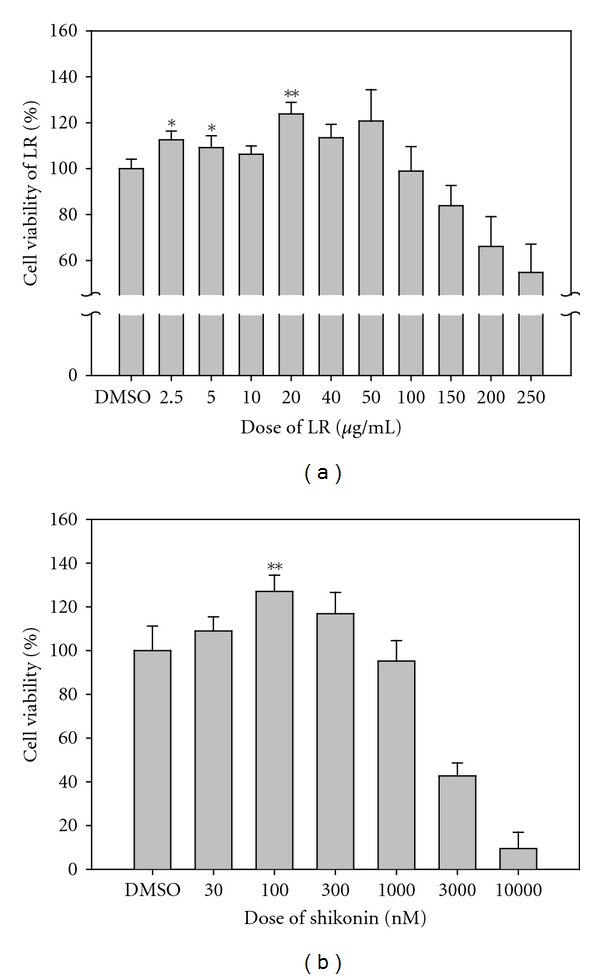
Effects of LR extracts and shikonin on human fibroblast cell viability. The graphs represent the ratio of viability of fibroblast cells treated with various concentrations of LR extracts or shikonin using cells treated with DMSO as the control. (a) LR extract was toxic to fibroblasts in a dose-dependent manner at higher concentrations and reaches the IC50 at the concentration of 250 *μ*g/mL. In contrast to the above toxicity, concentrations lower than 50 *μ*g/mL promoted cell viability. It should be noted that 20 *μ*g/mL of LR extracts were the optimal concentration for promoting cell viability, which gave an increase in the cell viability of more than 25%. Moreover, shikonin had a similar effect on cell viability (b), and the IC50 for fibroblasts was found to be about 3000 nM for shikonin. It was found the viability of fibroblasts was increased by >20% at 100 nM shikonin. Fibroblasts were seeded into a 24-well plate using 4 × 10^4^ cells per well. After the cells had attached, the designated drug concentration was used to treat the cells for 24 h. Cell viability was evaluated by the WST-1 assay, and the cell density was measured by spectrophotometer at OD450-OD690. It should be noted that fibroblasts treated with 0.5% of DMSO was used as the control. The Student's  *t*-test was used to evaluate the statistical significance of the results, which is presented as mean ± SD (*n* = 5). *P < 0.05; **P < 0.01, compared with the control.

LR extracts were generated to carry out the cell viability experiments. LR extracts were dissolved in DMSO and 0.5% DMSO in culture medium served as the control. Different concentrations of LR extracts were used to treat fibroblasts for 24 h, and the cell viability was measured by WST-1 assay, and the results monitored by spectrophotometry. As shown in Figure 1(a), cell viability was decreased in a dose-dependent manner and the IC50 was 250 *μ*g/mL. However, when the concentration was lower than 100 *μ*g/mL, the fibroblasts showed greater cell viability than the DMSO control. Based on this result, concentrations of LR extract lower than 100 *μ*g/mL were used to test the efficacy of LR for the viability of the fibroblast cell growth. It was found that viability of fibroblast cells was 25% greater than that of the control when the cells were treated with 20 *μ*g/mL of LR extract (Figure 1(a)). Similarly, it was found that the IC50 of fibroblast treated with shikonin (Figure 1(b)) was about 3000 nM and that 100 nM shikonin was able to significantly increase cell viability by more than 20%. Our HPLC analysis indicated that shikonin content in 20 *μ*g/mL LR extract is 43 nM (see Supplementary Figure 1 available online at doi:10.1155/2012/508972). Obviously these results demonstrate that shikonin alone has a smaller effect on cell survival than that of LR extract. These results seem to suggest that other effective components contained in the LR extract together with shikonin may have a synergistic effect on cell survival. Based on these observations, we chose to compare an LR extract of 20 *μ*g/mL (LR20), an LR extract of 5 *μ*g/mL (LR5), and shikonin at 100 nM (S100) during further experiments.

### 3.2. The Identification of Differentially Expressed Proteins in Fibroblast Treated with LR Extracts or Shikonin

**Figure 2:**
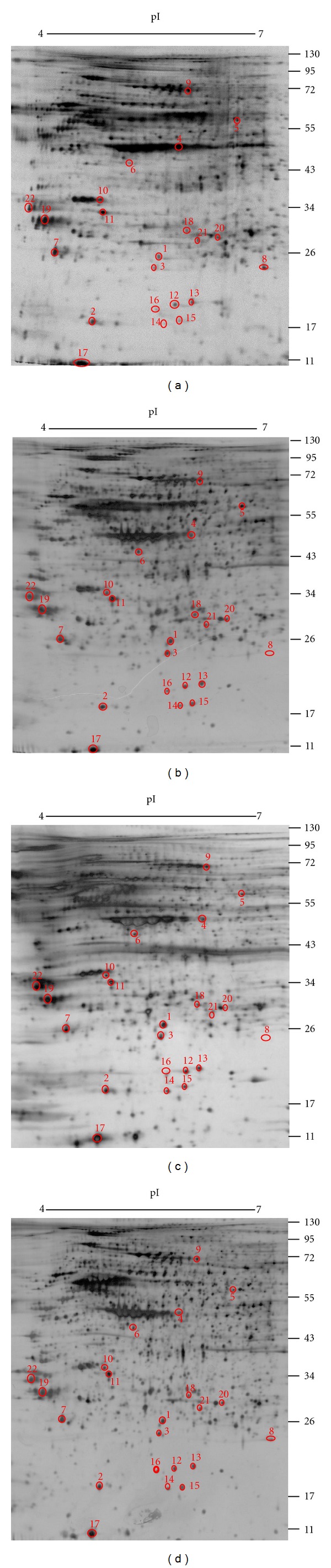
2D PAGE analysis of the protein differentially expressed in human fibroblast after treatment with the LR extracts or shikonin. Fibroblasts were treated with 5 *μ*g/mL LR extract, 20 *μ*g/mL LR extract, or 100 nM shikonin for 24 h, and then the whole cell lysates were collected and analyzed by 2D PAGE using a pH 4–7 IEF strips and 12.5% SDS-PAGE. The resulting gel was then visualized by silver staining. The treatments of the cells for the 2D PAGE are indicated below each gel and are (a) 0.5% of DMSO, (b) 5 *μ*g/mL LR extract (LR5), (c) 20 *μ*g/mL LR extract (LR20), and (d) 100 nM of shikonin (S100). It should be noted that there were 22 differentially expressed protein spots with ratio >1.5 or <0.8 fold compared to the DMSO control that were identified from LR5-, LR20-, and S100-treated cells. The corresponding protein spot identities are shown in Table 1.

**Figure 3:**
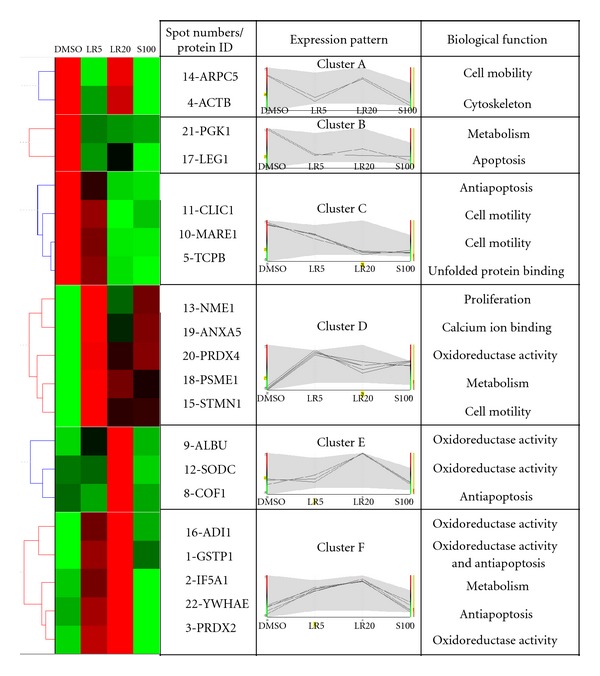
Hierarchical clustering and functional classification of protein expression induced in fibroblasts by the various drug treatments. The expression patterns of the identified proteins were categorized by UPGMA using Hierarchical Clustering Explorer 3.5 software, and the biological function classification was determined using BGSSJ and the SwissProt protein sequence database. Proteins with similar expression patterns were categorized into six different groups (clusters A, B, C, D, E, and F) in a tree-like diagram. Each row in the color mosaic map indicates one protein with a number matching the 2D-PAGE (Figure 2) and each column represents different groups of proteins identified from the fibroblasts treated with DMSO, LR5, LR20, and S100. A bright red color represents a high protein expression value and a bright green color represents a low protein expression value. Black color indicates that the protein was expressed at an average level. Further information on the differentially expressed protein spots is presented in Table 1.

In order to elucidate the molecular basis of the effect of the two LR extracts and shikonin on fibroblasts, the cells were treated with LR5, LR20, and S100 for 24 h, and cell lysates were collected and subjected to two-dimensional polyacrylamide gel electrophoresis (2D-PAGE) in order to display their protein profiles (Figure 2). It was possible to pinpoint 22 differentially expressed proteins and to carry out mass spectrometry on them (Table 1). In order to facilitate the functional analysis of the differentially displayed proteins, hierarchical functional clustering of the 22 proteins in response to various drug treatments was performed by HCE3.5 and BGSSJ (Figure 3). A total of six clusters (Figure 3 clusters A to F) were classified and nine groups of proteins with discrete biological functions (cell mobility, cytoskeleton, metabolism, apoptosis, calcium ion binding, unfolded protein binding, proliferation, oxidoreductase activity, and antiapoptosis) were identified. The pattern of protein clustering indicated that six proteins (1-GSTP1, 3-PRDX2, 9-ALBU, 12-SODC, 16-ADI1, and 20-PRDX4), which are involved in antioxidant activity, were all upregulated in fibroblast cells after LR20 treatment (Figure 3, clusters D, E, and F). Furthermore, LR20 treatment also induced three proteins (1-GSTP1, 8-COF1, and 22-YWHAE) that are involved in anti-apoptosis (Figure 3, clusters E and F). In addition, two proteins that are involved in cell mobility (10-MARE1 and 11-CLIC1) were downregulated by all three treatments in cluster C (LR5, LR20, and S100 treatments), indicating that these treatments might negatively regulate cell movement. It is probable that the induced antioxidant, anti-apoptosis, and cell mobility effects might be the dominant areas involved in the wound healing process of fibroblasts.

### 3.3. Effect of LR Extracts and Shikonin on Antiapoptotic and Antioxidative Activity

**Figure 4:**
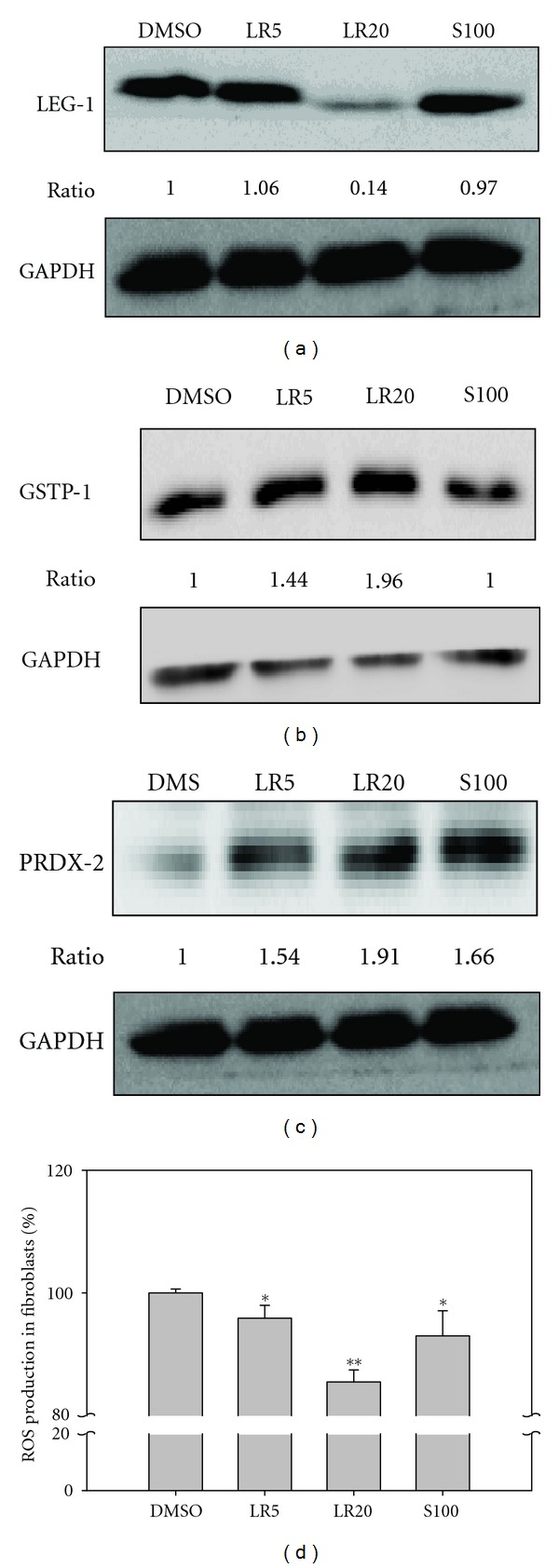
Immunoblotting of LEG-1 from the treated fibroblasts and the measurement of ROS within the treated fibroblasts. The immunoblot using LEG-1 antibody showed that LEG-1 (spot 17) was only significantly downregulated in cells that had undergone the LR20 treatment (a). On the other hand, GSTP-1 (B, spot 1, Figure 3, cluster F) and PRDX-2 (C, spot 3, Figure 3, cluster F) were upregulated in cells that underwent either LR treatment, which was confirmed by immunoblotting with specific antibodies. However, only overexpression of PRDX-2, but not overexpression of GSTP-1, was detected in cells after S100 treatment (c). The ROS assay showed that ROS production was significantly reduced in cells treated with LR20 (d). GAPDH was employed as the sample loading control. The related expression levels were detected by Fujifilm Multigauge ver. 2.0 and a Student's  *t*-test was used to evaluate the statistical significance. Results are from triplicate experiments and are presented as the mean ± SD (*n* = 3). *P < 0.05; **P < 0.01.

The apoptotic protein, galectin-1 (17-LEG1), was classified in cluster B and expression of this protein was downregulated in fibroblasts by both the two LR extracts and S100 treatment. However, immunoblotting showed that only LR20 was able to significantly inhibit the expression of LEG-1 (Figure 4(a)). On the other hand, the protein levels of 1-GSTP1 and 3-PRDX2 (antioxidant activity, cluster F) were enhanced by both LR treatments; however, S100 was only able to enhance the expression of 3-PRDX2 (Figures 4(b) and 4(c)). Furthermore, it is noteworthy that LR20 was able to downregulate the ROS content of up to 15% of fibroblast cells, which is the greatest decrease among all of the treatments (Figure 4(d)). Thus, both the immunoblotting and bioassay results agree that the LR extracts are able to significantly downregulate the ROS content of fibroblasts in a dose-dependent manner.

### 3.4. Effect of LR Extracts and Shikonin on Cell Movement Ability

**Figure 5:**
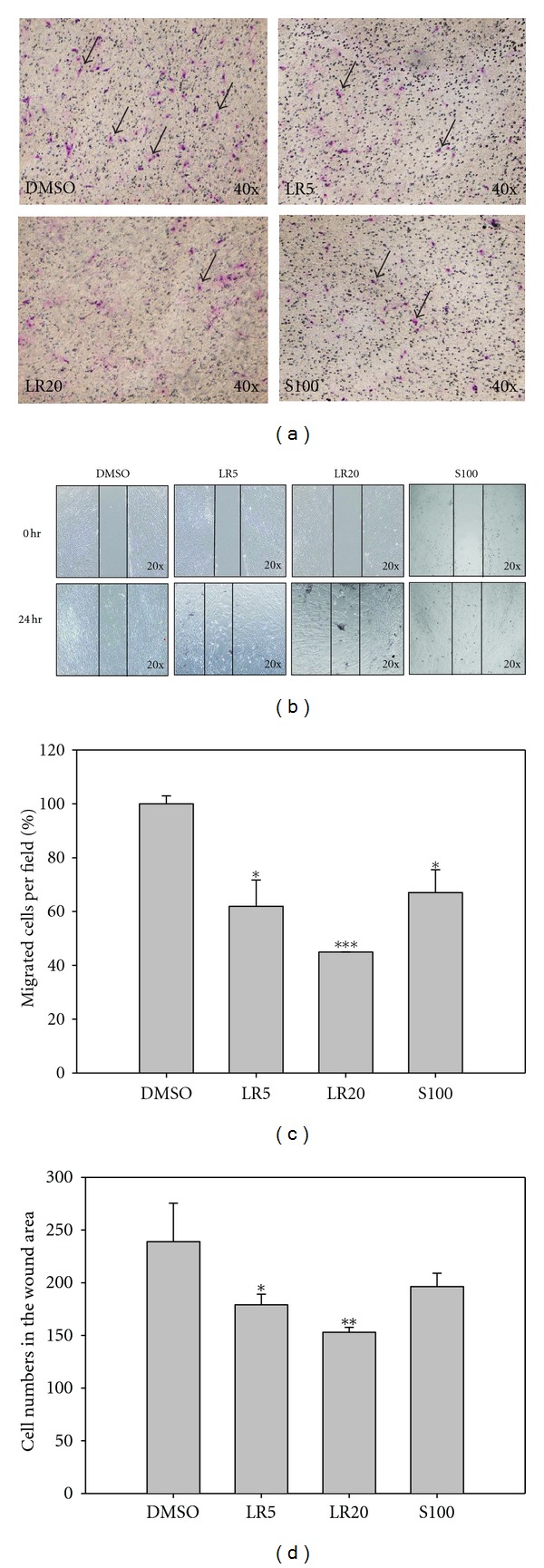
The Boyden chamber migration test and the wound healing assay were used to detect the motility of fibroblasts after the various drug treatments. The migration assay showed that LR5-, LR20-, and S100-treated cells underwent less migrated through the chamber (a). The wound healing assay further demonstrated that treatment with the LR extracts was able to significantly decrease the migration of cells in a dose-dependent manner (b). Furthermore, the active component S100 was also able to create a similar effect on cell migration. Quantification of the chamber migration (c) indicated that LR20 significantly reduced cell migration and to the largest extent. Quantification of the wound area (d) showed that fewer cells migrated into the wound area when the cells were treated with LR20. The results are expressed as mean ± SD (*n* = 3). *P < 0.01; ***P < 0.001, compared with DMSO.

11-CLIC1 and 10-MARE1 are the two proteins that are involved in cell mobility and these were downregulated by both of the LR extracts and by shikonin treatment (Figure 3, cluster C). The Boyden chamber migration assay showed that (Figures 5(a) and 5(c)) fewer cells migrated through the Transwell after LR5, LR20, and S100 treatments compared with DMSO. Overall, the LR20 treatment seemed to be the most effective and reduced migration by about 55%. Furthermore, the wound healing assay showed a similar effect on the inhibition of cell migration into the wound area (Figures 5(b) and 5(d)). The results demonstrated that LR extracts and its active component, shikonin, are able to significantly decrease cell movement.

### 3.5. Effect of LR Extracts on Collagen Secretion and TGF-*β* Expression

**Figure 6:**
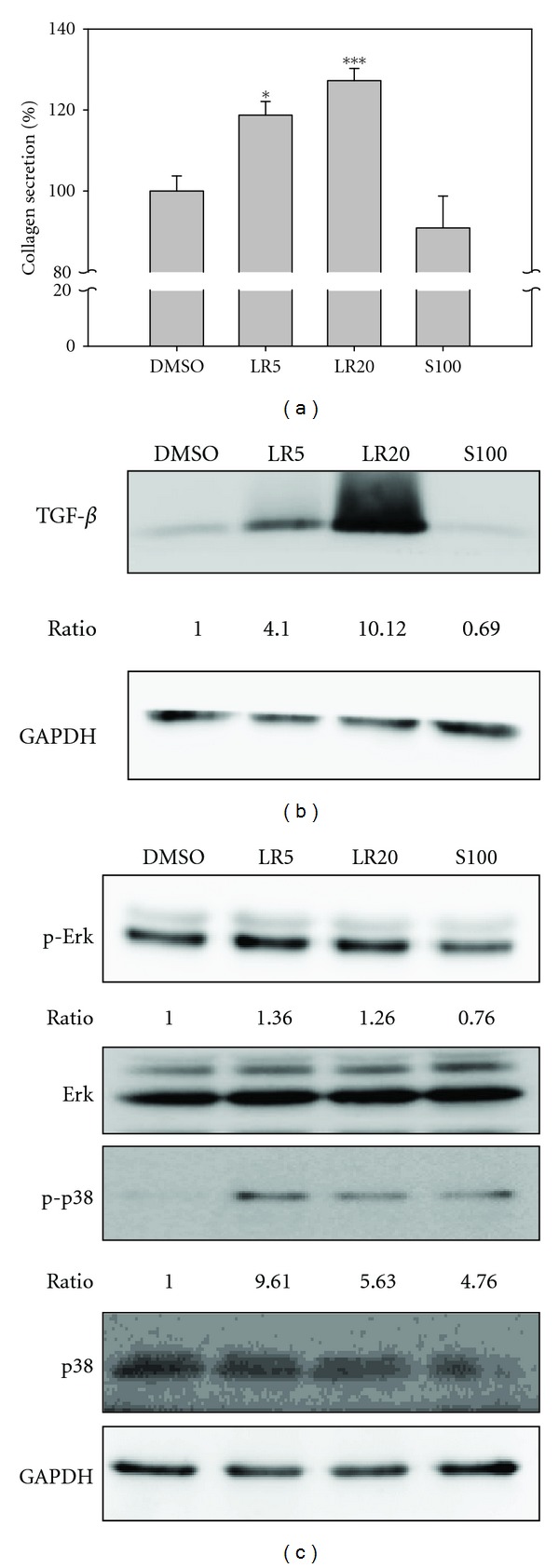
Detection of collagen secretion, TGF-*β* expression, and other related cell signals. LR5 and LR20 treatments are able to significantly upregulate collagen secretion by fibroblasts in a dose-dependent manner (a). It was also found that the upregulation of collagen was concomitant with the overexpression of TGF-*β* (b). It was further confirmed that p-Erk and p-p38 were also upregulated in cells after LR5 and LR20 treatments (c). In contrast, the expression of p-Erk and p-p38 was downregulated in S100-treated fibroblasts. The ratios of p-Erk and p-p38 were normalized against GAPDH and then compared with DMSO using the Fujifilm Multigauge system. The experiments were carried out in triplicate and showed the same results. *P < 0.01; ***P < 0.001.

 Collagen secretion plays an important role in the wound healing process, and the expression of this protein is positively regulated by TGF-*β* [11]. The result of the Sircol collagen assay showed that LR5 and LR20 were able to significantly increase collagen secretion in a dose-dependent manner (Figure 6(a)). Furthermore, immunoblotting also confirmed that LR extracts, but not shikonin, were able to dramatically increase TGF-*β* expression (Figure 6(b)), which would promote the secretion of collagen. 

 TGF-*β* signaling not only induces collagen secretion via SMADs, but also this occurs via SMAD-independent pathways such as MAPK and Akt, which are involved in cell proliferation [12]. Immunoblotting showed that expression of p-Erk was increased after LR5 and LR20 treatments (Figure 6(c)). Furthermore, expression of p-p38 was also found to be increased after treatment with both LR extracts and after S100 treatment. These findings suggest that LR extracts may target the MAPK signaling pathway in fibroblast cells, which would benefit cell proliferation during the wound healing process.

### 3.6. Effect of LR Extracts on the Wound Healing Speed *In Vivo*

**Figure 7:**
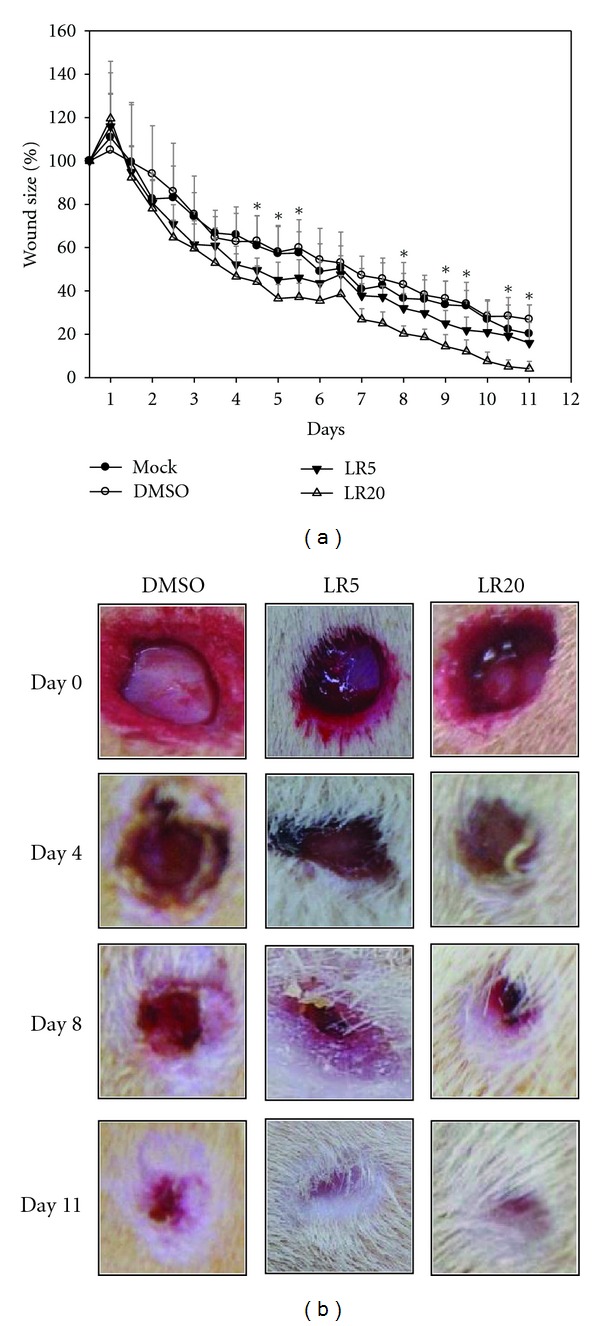
SD rat experiment to verify the efficacy of the LR extracts in wound healing. The diameter of the wounds was used as a guideline for the rate of wound healing. Two wound circles were generated on the back of SD rats using an 8 mm biopsy punch. DMSO, LR5, and LR20 were used to treat the wounds and the diameter of the wounds was measured twice per day. It was found that the efficacy of wound closing rate of the wounds after LR5 and LR20 treatments was far much greater that when the wounds were treated with DMSO only (a). The wound healing rate was also recorded by digital camera at interval of 4 days (b). This demonstrated that the wound started to shrink after 2 days and continued to shrink further until 11 days. It is worth noting that wounds treated with LR20 showed the fastest wound closing rate compared to the other treatments (b). Results are triplicates and are presented as means ± SD (*n* = 6). *P < 0.05; **P < 0.01.

The effect of LR extracts on the wound healing speed was confirmed by an *in vivo* test. Two 8 mm biopsy punch wounds were created on the flank back of SD rats, and then the wounds were treated with DMSO or either LR extract twice per day. The results showed that after 2 days of treatment, the size of the wounds in the SD rats treated with either of the LR extracts was significantly decreased when compared with DMSO-treated SD rats and the mock control (Figure 7(a)). After LR treatment for 11 days, it was observed that the healing process of the wound was improved day by day. The healing process was captured by digital camera once every 4 days after treatment (Figure 7(b)). This photographic record clearly demonstrated that treatment with LR extract was able to significantly accelerate wound healing speed *in vivo*, which agrees with the above *in vitro* findings.

## 4. Discussion

The molecular basis of the wound healing ability of a single herb, Lithospermi Radix (LR) and its major active component, shikonin, was studied using a proteomic approach. Apparently both LR and shikonin are able to promote cell viability, enhance antioxidant capacity, and downregulate cell mobility of fibroblasts. However, the biological functioning of the LR-treated fibroblasts shows that LR has some advantages over shikonin in terms of antiapoptosis, collagen secretion, and TGF-*β* expression. A previous report has revealed that skin fibroblast plays an important role in the proliferation phase of wound healing and is responsible for regeneration at the wound margin [13]. Therefore, exploring the molecular basis of how LR promotes wound recovery via its effect on fibroblasts during the wound healing process is of both academic and clinical interest.

 2D-PAGE and mass spectrometry analysis is the most powerful state-of-the-art technology available for revealing the coordination between protein function and the responses of fibroblast cells during the wound healing processes. A previous study has reported that proteins with related functions are often coordinately expressed [14]. Similarly, we found in this work that differentially displayed proteins with related functions can be categorized into discrete hierarchical clusters (Figure 3). In total, 22 differentially displayed proteins were identified as present in fibroblast cells in response to LR or shikonin treatment by the present study and these could be categorized into six hierarchical clusters (Figure 3). The detailed discussion of these categories of proteins in relation to how fibroblasts function during the wound healing process is outlined below.

### 4.1. Protein Functions Involved in Antioxidant Activity

Reactive oxygen species (ROS) were very important during the wound healing process. During the inflammatory phase, immune cells such as neutrophils and macrophages infiltrate into the wounded site and produce large amount of ROS that protect against foreign bacteria and fungi [15]. In addition, fibroblasts can be induced by proinflammatory cytokines to produce ROS [16]. Therefore, after the inflammatory phase of healing process, cells are able to regulate themselves and express ROS-detoxifying enzymes and antioxidant proteins [17], which result in an attenuation of ROS induced damage. 

We found that peroxiredoxin 2 (3-PRDX2) and glutathione-S transferase Pi (1-GSTP1) were upregulated in cells treated with LR (Figure 3, cluster F). Furthermore, while superoxide dismutase (12-SODC) was only upregulated after LR20 treatment (Figure 3, cluster E), both LR and S100 treatment were able to upregulate 20-PRDX4 expression (Figure 3, cluster D). Shukla et al. [18] have demonstrated that without free radical scavengers like SODC and GST, healing is partially or completely impaired. Furthermore, application of SOD hydrogels to wounds in rats is able to increase the wound healing rate [19]. The PRDXs are a multifunctional family with six members, and they have been reported to act as antioxidant thioredoxin-dependent peroxidases [20]. Our results also demonstrated that the ROS content of fibroblast after LR20 treatment was significantly decreased in a dose-dependent manner. Thus, these results suggest that LR20-treated fibroblast has improved the antioxidant activity compared to S100 which will give greater protection of the cells against ROS damage.

### 4.2. Protein Functions Involved in the Regulation of Apoptosis

Galectin-1 (17-LEG1) was downregulated by LR and S100 treatment (Figure 3, cluster B), while glutathione S transferase Pi (1-GSTP1) and 14-3-3 protein epsilon isoform (22-YWHAE) were upregulated by LR treatment (Figure 3, cluster F). In addition, cofilin-1 (8-COF1) was upregulated only by LR20 treatment (cluster E). It has been reported that LEG1 is a *β*-galactoside ligand that it is able to inhibit cell proliferation and promote cellular apoptosis in normal cells [21]. GSTP1 overexpression in HEK293 has been shown to inhibit pro-caspase3 activation and MEKK1-mediated apoptosis [22]. YWHAE is an isoform of 14-3-3 protein, and it was found to be upregulated during photoaging and in intrinsically aged human skin [23]. Furthermore, YWHAE has been demonstrated to protect cells from UV-induced apoptosis [24]. COF1 has been reported to prevent apoptosis that is induced by oxidative stress during inflammation [25]. Our results demonstrated that LR20 is able to downregulate LEG1 and upregulates GSTP1, YWHAE, and COF1, which should result in the prevention of fibroblast apoptosis. Obviously, the antiapoptotic effect of LR20 on fibroblast cells ought to improve the cell survival rate of fibroblasts and this should be achieved to a greater extent than with S100 treatment.

### 4.3. Protein Functions Involved in Collagen Secretion and Cell Mobility

Chloride intracellular channel protein 1 (11-CLIC1) and microtubule-associated protein RP/EB family member 1 (10-MARE1) were both downregulated by LR20 and S100 treatments (cluster C). Furthermore, stathmin (15-STMN1) was upregulated after LR and S100 treatments (cluster D). CLIC1 has been reported to be upregulated in the highly metastatic cell line GBC-SD18H compared to less metastatic cell line GBC-SD18L [26]. Furthermore, when the expression of CLIC1 in Hca-F was silenced, cell proliferation and invasion was decreased [27]. STMN1 has been shown to act as microtubule-destabilizing protein and interact with p27 to coordinate cell mobility [28]. 

Collagen secretion and cell mobility are two antagonistic effects that are both important to the wound healing process. Collagenase-1 activity is required for keratinocyte migration on collagen matrix [29]. Furthermore, fibroblast migration also requires collagenase to enable them to move within a wound [30]. In this work, the proteomic analysis, the migration assay, and the wound healing assay all point towards the migration ability of fibroblasts being significantly downregulated by LR and S100 treatments. However, in the LR-treated fibroblasts, increased collagen expression was found to be concomitant with overexpression of the TGF-*β* (Figure 6(a)). In contrast, in the S100-treated fibroblasts, collagen secretion and the TGF-*β* expression were found to be both downregulated. These experimental results demonstrate that LR may be more effective as a wound healing agent with respect to fibroblast proliferation and retention in the wounded region.

### 4.4. Protein Functions Involved in Metabolism or Cell Proliferation

Phosphoglycerate kinase 1 (21-PGK1) was downregulated by LR and S100 treatments (cluster B), while proteasome activator complex subunit 1 (18-PSME1), also called REGalpha or PA28alpha, was upregulated by LR and S100 treatments (cluster D). PGK1 is known to be a metabolic enzyme involved in glycolysis. Furthermore, it has been recently discovered that PGK1 might be a protein biomarker for the cell's intracellular oxidative status [31, 32]. PSME1 is known to be a proteasome activator that binds to the proteasome and stimulates peptide hydrolysis [33]. Recent research has shown that PSME1 is not only able to enhance the cell's ability to remove misfolded and oxidized proteins, but it is also able to protect cardiomyocytes from oxidative stress [34]. Eukaryotic translation initiation factor 5A-1 (2-IF5A1) was upregulated by LR treatment (cluster F) and has been demonstrated to be a proliferation biomarker in vulvar high-grade intraepithelial neoplasia (VIN) [35]. Moreover, depletion of IF5A1 seems to lead to G1 arrest [36]. Nucleoside diphosphate kinase A (13-NME1, also named NM23) was upregulated by LR and S100 treatments (cluster D). This protein is multifunctional and seems to be able to promote cellular proliferation and differentiation in response to environment factors [37]. Furthermore, NME1 has been shown to be a novel regulator of epidermal homeostasis, which is associated with the regulation of proliferation, differentiation, and survival of keratinocytes [38].

 LR20 and S100 were found to be the concentrations giving the highest viability for fibroblast cells (Figure 1) and this agrees with the results of our proteomic analysis whereby NME1 and IF5A1 are upregulated by these treatments, which should promote cell proliferation (Figure 3, clusters D and F). Furthermore, when fibroblasts are treated with LR and S100, phosphorylated-p38 and phosphorylated-Erk were found to be upregulated, which supports the hypothesis that this treatment does indeed promoted the proliferation of treated fibroblasts.

### 4.5. Potential Molecular Evidence for the Involvement in the Wound Healing Process of LR Ethanol Extracts and Their Active Component Shikonin

**Figure 8:**
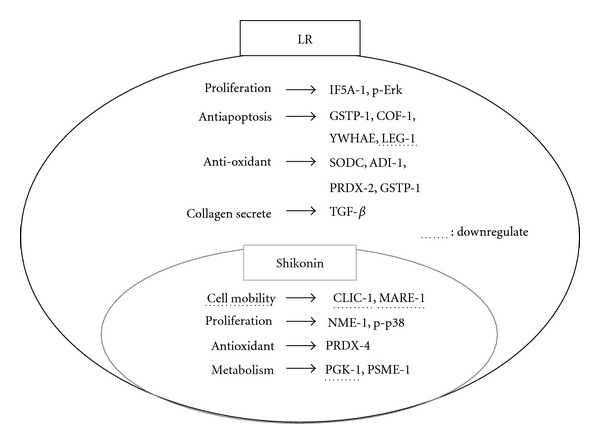
Comparison of LR and shikonin-induced differential expression of proteins in fibroblasts involved in wound healing processes. Only the differentially expressed proteins induced by either LR or shikonin are shown. Note that the proteins underlined with broken lines represent downregulated proteins, otherwise the proteins are upregulated. It is worth noting that the differentially expressed proteins induced by shikonin form a subset of all the differentially expressed proteins induced by LR. Thus, all differentially expressed proteins placed in the small circle represent the proteins induced by shikonin.

Taken together, our results reveal that a possible pathway of the wound healing process is induced by LR ethanol extracts and their active component shikonin (Figure 8). Both LR and S100 seem to inhibit cell mobility (10-MARE1 and 11-CLIC1), to promote cell proliferation (13-NME1 and p-p38), to produce antioxidant activity (20-PRDX4), and to coordinate metabolism (21-PGK1 and 18-PSME1). Nonetheless, LR does induced many extra functionally useful proteins that are barely affected by S100; these proteins are involved in proliferation (2-IF5A1 and p-Erk), antiapoptosis (1-GSTP1, 8-COF1, 17-LEG1, and 22-YWHAE), antioxidant activity (1-GSTP1, 3-PRDX2, 12-SODC, and 16-ADI1), and collagen secretion (TGF-*β*).

Lithospermi Radix and *Angelica sinensis* (AS) are the two major components of Shiunko, a traditional Chinese medicine formula. In comparison of this work with our previous study [8], it is clear that the wound-healing promoting mechanisms of AS and LR are different to some extent. For example, AS is able to promote cell mobility by inhibiting the expression of NDKB and VIME. However, LR reduces cell mobility by inhibiting the expression of CLIC1. Moreover, AS treatment of cells promoted cell viability by upregulating CAPNS1 and p-Akt and inhibited LEG1 expression. However, LR treatment promoted cell viability by upregulating NME1, IF5A1, and p-p38. On the other hand, the effects of LR and AS treatment were similar for some proteins. Antioxidant ability was increased by upregulating of the PRDX family and GSTP1 after both treatments. Similarly, there was also upregulation of p-Erk by both treatments, which would lead to increased cell proliferation. Interestingly, when fibroblasts were treated with LR, there was a greater induction of collagen secretion when they were treated with AS, with the upstream signal TGF-*β* also showing the same trend. These results show that both LR and AS are able to promote the wound healing process, but do so in different ways in some cases. Nonetheless, the combination of these two herbal medicines together is likely to produce improved coordination of the wound healing process and enhances its efficacy synergistically. 

 In conclusion, a proteomic platform was used to elucidate the possible molecular basis of the wound healing process induced by LR extracts and shikonin with respect to fibroblasts. This work provides a feasible and effective tool that can be used for the study of the molecular basis of traditional herbal medicines.

## Supplementary Material

Supplementary Figure 1: The content of the LR extracts as detected by HPLC chromatography. LR has an absorbance peak at 520 nm. The retention time of shikonin is 12.6 min. (A) shikonin standard. (B) ethanol extract of LR. The concentration of shikonin in the LR extract was determined to be 0.62 mg/g.

## Figures and Tables

**Table 1:** Proteins showing altered abundance after treatment with either the LR extracts or shikonin compared to the DMSO control and identified by LC-MS/MS.

ID	Protein identity	Accession number	Abbreviation	MW/pI	Score	Match peptide	Sequence coverage (%)	LR 5/DMSO	LR 20/DMSO	S100/DMSO
1	Glutathione S-transferase P	P09211	GSTP1	23/5.43	224	8	46	↑ 2.26	↑ 2.66	↑ 1.61
2	Eukaryotic translation initiation factor 5A-1	P47942	IF5A1	17/5.08	58	4	23	↑ 1.17	↑ 1.30	↓ 0.96
3	Peroxiredoxin-2	P32119	PRDX2	22/5.38	143	6	23	↑ 1.65	↑ 1.84	↓ 0.89
4	Actin, cytoplasmic-1	P60711	ACTB	42/5.29	136	4	8	↓ 0.44	↓ 0.89	↓ 0.22
5	T-complex protein 1 subunit beta	P78371	TCPB	36/8.43	169	12	21	↓ 0.88	↓ 0.68	↓ 0.66
6	Glutaredoxin-3	O76003	GLRX3	37/5.31	81	8	19	↓ 0.61	↓ 0.69	↓ 0.83
7	Translationally controlled tumor protein	P13693	TCTP	20/4.84	119	4	17	↓ 0.66	↓ 0.40	↓ 0.39
8	Cofilin-1	P23528	COF1	18/8.22	113	7	24	↓ 1/∞	↑ 9.67	↓ 1/∞
9	Serum albumin	P02768	ALBU	69/5.92	119	2	3	↑ 1.24	↑ 1.78	↑ 1.04
10	Microtubule-associated protein RP/EB family member 1	Q15691	MARE1	30/5.02	65	3	17	↓ 0.85	↓ 0.61	↓ 0.61
11	Chloride intracellular channel protein 1	O00299	CLIC1	27/5.09	114	3	12	↓ 0.89	↓ 0.64	↓ 0.69
12	Superoxide dismutase	P00441	SODC	16/5.70	61	1	9	↑ 1.02	↑ 1.54	↓ 0.91
13	Nucleoside diphosphate kinase A	P15531	NME1	17/5.83	63	2	5	↑ 1.20	↑ 1.30	↓ 0.92
14	Actin-related protein 2/3 complex subunit 5	O15511	ARPC5	16310/5.47	179	6	27	↓ 0.51	↓ 0.97	↓ 0.49
15	Stathmin	P16949	STMN1	17292/5.76	134	6	36	↑ 2.54	↑ 1.98	↑ 1.99
16	1,2-Dihydroxy-3-keto-5-methylthiopentene dioxygenase	Q9bv57	ADI1	21484.6/	2.5 (criteria)	4	22	↑ 1.75	↑ 2.21	↑ 1.22
17	Galectin-1	P09382	LEG1	14706/5.34	81	3	19	↓ 0.39	↓ 0.55	↓ 0.28
18	Proteosome activator complex subunit 1	Q06323	PSME1	28705/5.78	289	7	27	↑ 3.12	↑ 2.64	↑ 2.37
19	Annexin A5	P08758	ANXA5	35914/4.94	2.5 (criteria)	16	65	↑ 2.31	↑ 1.68	↑ 1.98
20	Peroxiredoxin-4	Q13162	PRDX4	30521/5.86	105	4	14	↑ 2.81	↑ 2.27	↑ 2.51
21	Phosphoglycerate kinase 1	P00558	PGK1	44586/8.30	94	1	4	↓ 0.45	↓ 0.43	↓ 0.41
22	14-3-3 protein epsilon isoform	P62258	YWHAE	26504/4.76		n/a	n/a	↑ 1.86	↑ 1.31	↑ 1.58
