# Supplementary material for: The Molecular Basis of Wound Healing Processes Induced by Lithospermi Radix: A Proteomics and Biochemical Analysis
Source: Evid Based Complement Alternat Med. 2012 Sep 17;2012:508972. doi: 10.1155/2012/508972 (PMC3457683; doi:10.1155/2012/508972)
Supplement: Supplementary file 1 — Supplementary Figure 1: The content of the LR extracts as detected by HPLC chromatography. LR has an absorbance peak at 520 nm. The retention time of shikonin is 12.6 min. (A) shikonin standard. (B) ethanol extract of LR. The concentration of shikonin in the LR extract was determined to be 0.62 mg/g. [file 508972.f1.pdf]

Supplementary Fig.1

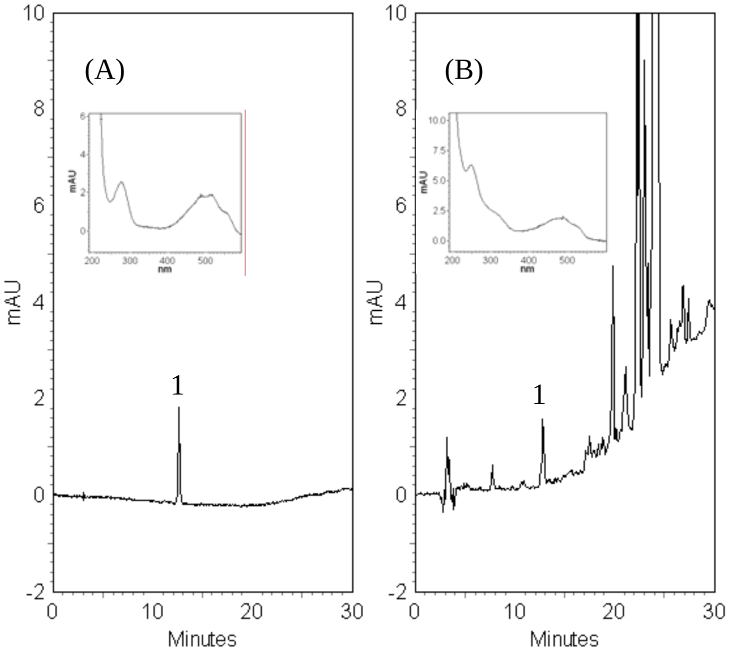

Fig.1 The content of the LR extracts as detected by HPLC chromatography. LR has an absorbance peak at 520 nm. The retention time of shikonin is 12.6 min. (A) shikonin standard. (B) ethanol extract of LR. The concentration of shikonin in the LR extract was determined to be 0.62 mg/g.
